# Reproducible asystole following vagal nerve stimulator lead replacement: a case report

**DOI:** 10.1186/s12883-022-02585-6

**Published:** 2022-03-04

**Authors:** Hayden Scott, Alexandra Moore, Hakan Paydak, Kelsey Hundley, Viktoras Palys

**Affiliations:** 1 College of Medicine, University of Arkansas for Medical Sciences, Little Rock, AR USA; 2 Department of Cardiology, University of Arkansas for Medical Sciences, Little Rock, AR USA; 3 Department of Neurosurgery, University of Arkansas for Medical Sciences, Little Rock, AR USA

**Keywords:** Seizure, Intractable epilepsy, Vagal nerve stimulation, Surgery, Revision, Asystole, Case report

## Abstract

**Background:**

Vagal nerve stimulation (VNS) is approved therapy for the treatment of intractable epilepsy. The stimulation of either nerve, left or right, is effective. However, due to the anatomic and physiological effects of cardiac innervation, the right vagus nerve is typically avoided in order to minimize the risk of cardiac bradyarrhythmias. The location of the VNS lead contacts on the nerve can also have an effect, namely, more distally placed contacts have been associated with lower risk of cardiac arrhythmias, presumably by avoiding vagal cervical cardiac branches; however, our case demonstrates reproducible asystole despite left sided, distal VNS lead placement.

**Case presentation:**

We report a 28-year-old male patient with pharmacoresistant generalized clonic-tonic seizures. The VNS therapy with 1.5 mA output and 16% duty cycle drastically reduced his seizure burden for several years. The breakthrough seizures along with stabbing pain episodes at the implantable pulse generator (IPG) site have prompted the VNS lead revision surgery with new lead contacts placed more caudally than the old contacts. However, the intraoperative device interrogation with 1 mA output resulted in immediate asystole for the duration of stimulation and it was reproducible until the output was decreased to 0.675 mA.

**Conclusions:**

Our case highlights the possibility of new severe cardiac bradyarrhythmias following surgical VNS lead replacements even in patients without preoperatively known clinical side effects. We suggest preoperative electrocardiography and cardiology consultation for detected abnormalities for all patients undergoing new VNS implantations, as well as revision surgeries for VNS malfunctions. Intraoperatively, the surgeon and anesthesia team should be vigilant of cardiac rhythms and prepared for the immediate management.

## Background

The World Health Organization estimates that more than 50 million individuals across the world are affected by seizure disorders. Fortunately, approximately two-thirds of the patients are able to achieve complete seizure control using pharmacologic therapies [1]. On the contrary, up to 30% of patients suffer with uncontrolled epilepsy [2]. Most literature has agreed on medically intractable (the other names – refractory, pharmacoresistant) epilepsy being defined as a failure to control seizures using two trials of appropriately chosen, dosed, and tolerated antiepileptic drugs. The high number of patients with intractable epilepsy leads to treatment attempts with more invasive options that include neuromodulation procedures.

The vagus nerve, also referred to as a tenth (X) cranial nerve, is largely responsible for the autonomic regulation of functions of the cardiovascular, respiratory, and gastrointestinal systems. Afferent fibers of the vagus nerve terminate in the nucleus tractus solitarius and project to various brain regions including the raphe nuclei, locus coeruleus, amygdala, hypothalamus, and thalamus [3].

Vagal nerve stimulation (VNS) for the treatment of intractable epilepsy became approved by the Food and Drug Administration (FDA) in the United States in 1997 [4]. It is worth notion, that VNS is also approved by FDA in 2007 for the treatment of drug-resistant depression.

During the VNS implantation procedure two platinum-iridium spiral contacts are coiled around the cervical portion of vagus nerve where nerve travels inside carotid sheath. Importantly, the negative contact is placed cranially and the positive contact is placed caudally on the nerve – this way the anodal block phenomenon limits the efferent stimulation and promotes action potential propagation towards the brain. The rest of lead is then tunneled subcutaneously into the infraclavicular position where it is connected to the implantable pulse generator (IPG) situated in the surgically created subcutaneous chest wall pocket on pectoralis fascia.

**Fig. 1:**
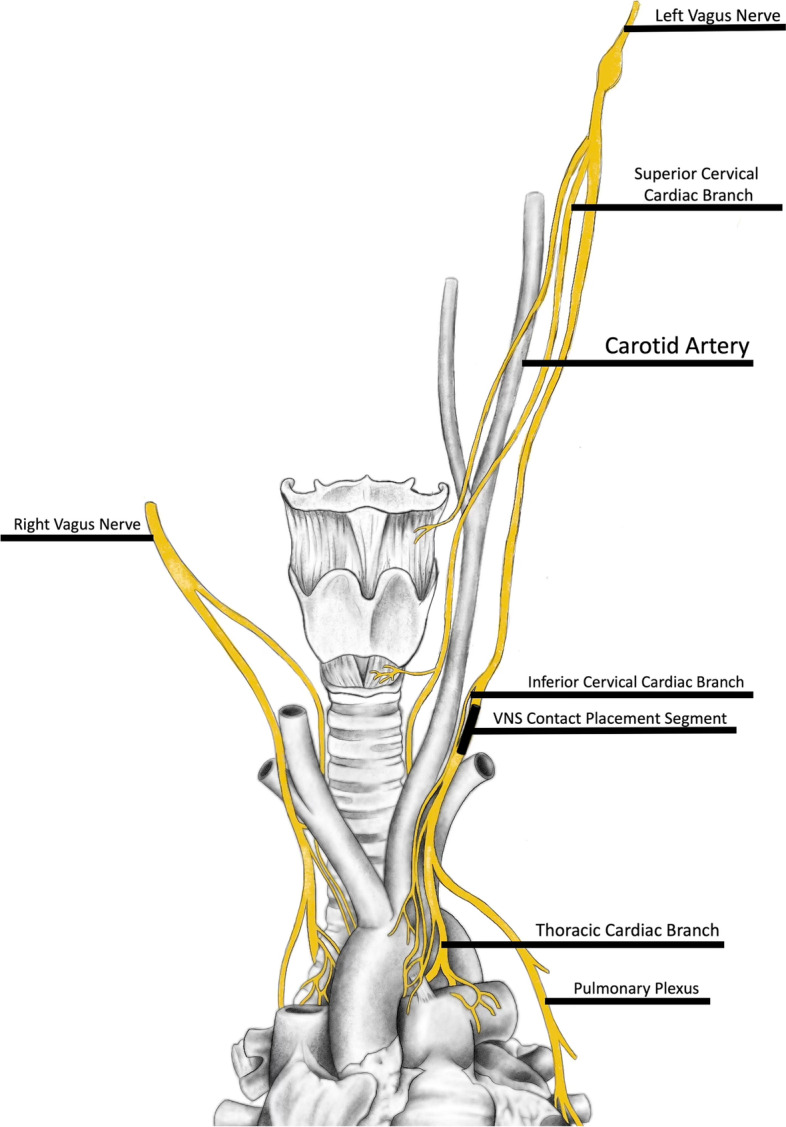
Schematic of vagal innervation. Anatomical illustration demonstrating cardiac vagal innervation

Anatomically, the right vagus nerve innervates the sinoatrial node, and the left vagus nerve—the atrioventricular node. Upon stimulation of the vagus nerve, the parasympathetic effects result in decreased heart rate, potentially resulting in symptomatic bradyarrhythmias up to atrioventricular blocks [5]. The stimulation of either nerve, left or right, is effective from epileptological standpoint. However, due to the anatomo-physiological specifics of cardiac innervation (Fig. 1), the right vagus nerve is typically avoided to help minimize the risk of cardiac arrhythmias [6]. Another surgical aspect to consider is the location, or height, of VNS lead contacts on the cervical vagus nerve, as it can also have an effect on cardiac complications. Notably, more distally placed contacts have typically been associated with lower risk of cardiac arrhythmias, presumably by avoiding vagal cervical cardiac branches; however, our case demonstrates reproducible asystole despite left sided, distal VNS lead placement. Additionally, one should be aware of the other vagus-look-alike nerves such as the phrenic nerve and ansa cervicalis as the inadvertent contact placement and/or stimulation of such nerves are also possible complications due to their anatomical proximity to vagus nerve. Unilateral phrenic nerve function interference is often asymptomatic at rest, but can result in dyspnea during exertion [7]. Ansa cervicalis stimulation may result in postoperative neck muscle contractions [8].

## Case presentation

**Fig. 2:**
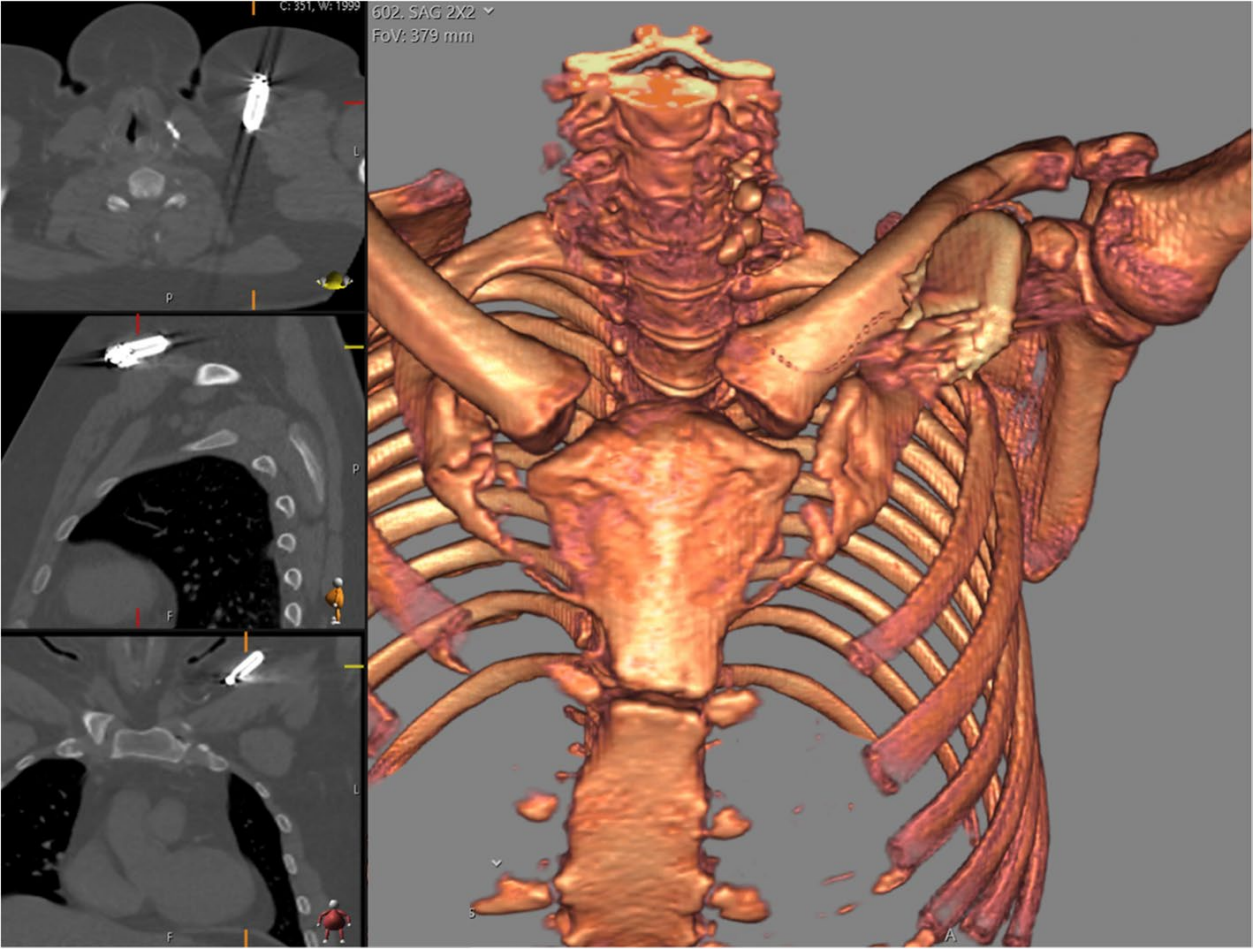
Preoperative CT of the neck and chest demonstrating oblique positioning of IPG without other VNS hardware or surrounding tissue abnormalities

**Fig. 3:**
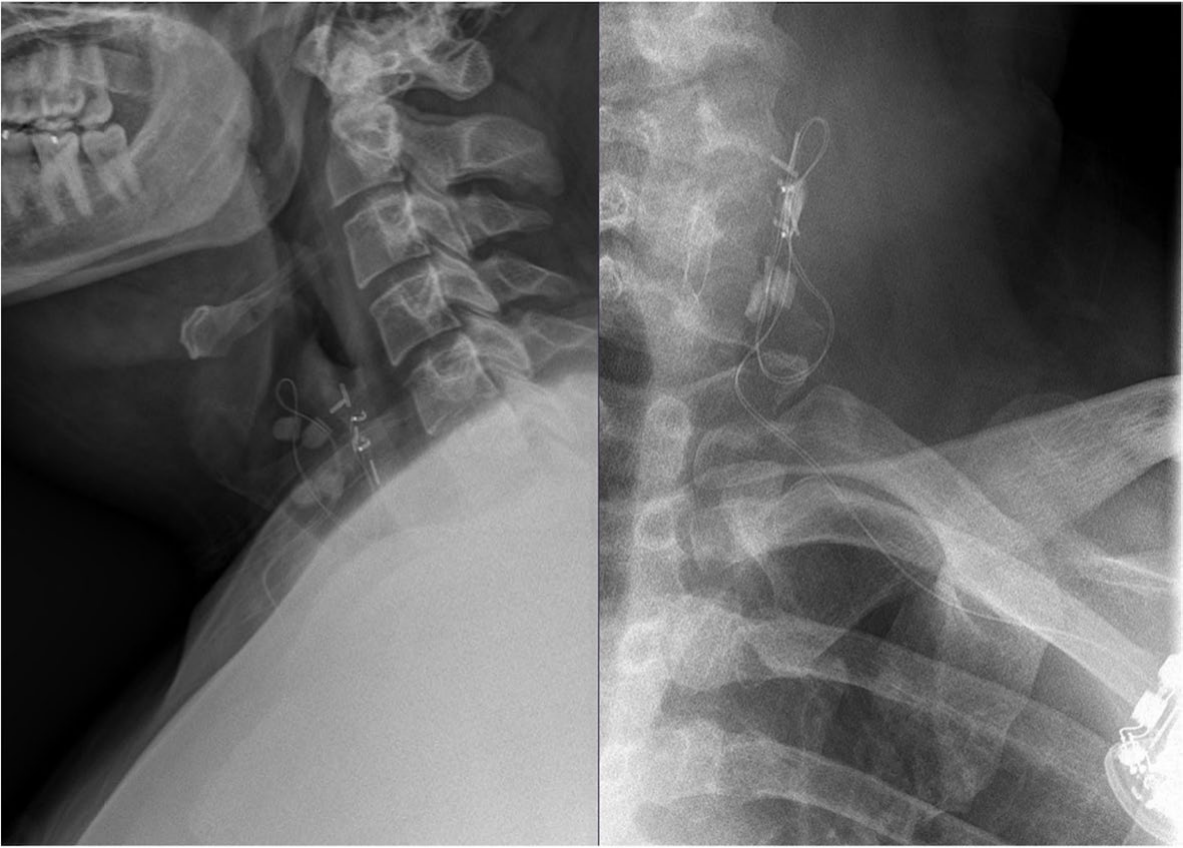
Preoperative plain radiograph of the neck demonstrating VNS electrode helical contacts at the C5 level and no visible VNS hardware discontinuity

**Fig. 4:**
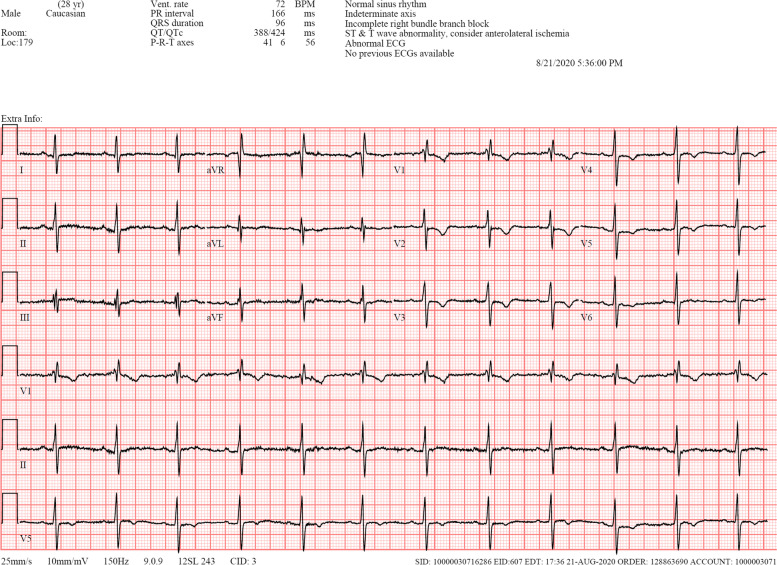
Preoperative ECG demonstrating incomplete right bundle branch block

**Fig. 5:**
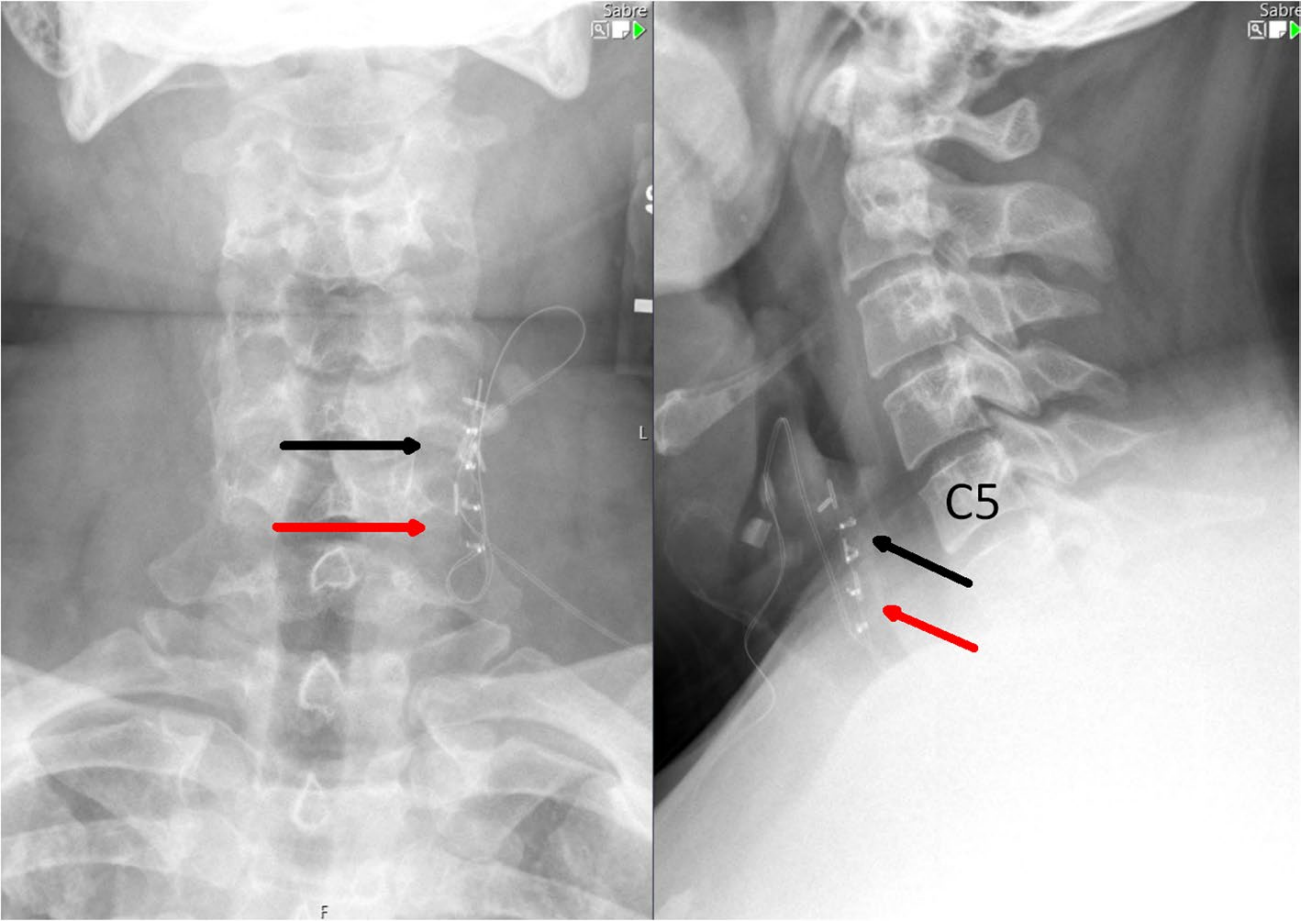
Postoperative plain radiograph of the neck demonstrating the retained old VNS electrode helical contacts (black arrows) at the C5 level as well as new VNS electrode helical contacts (red arrows) positioned more caudally

**Fig. 6:**
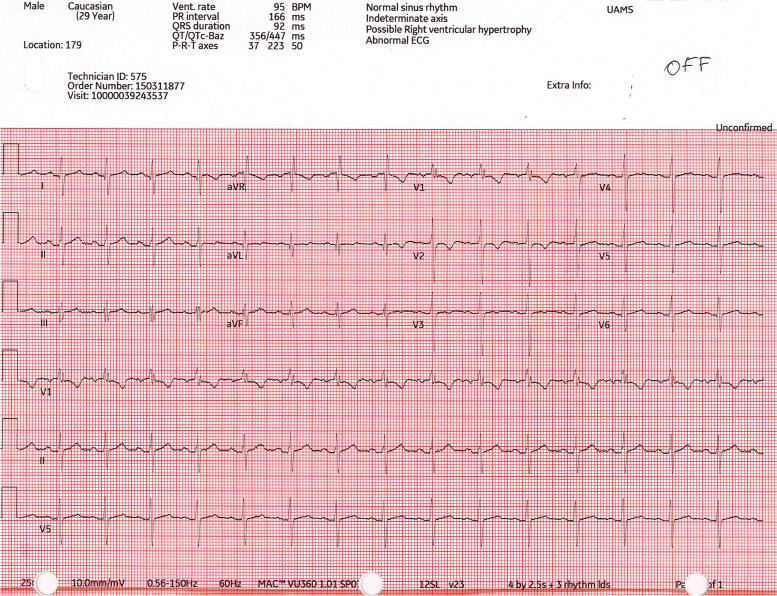
Postoperative ECG, 12 months after surgery - the normal VNS output was turned off (0 mA) and ECG was recorded to establish the baseline

**Fig. 7:**
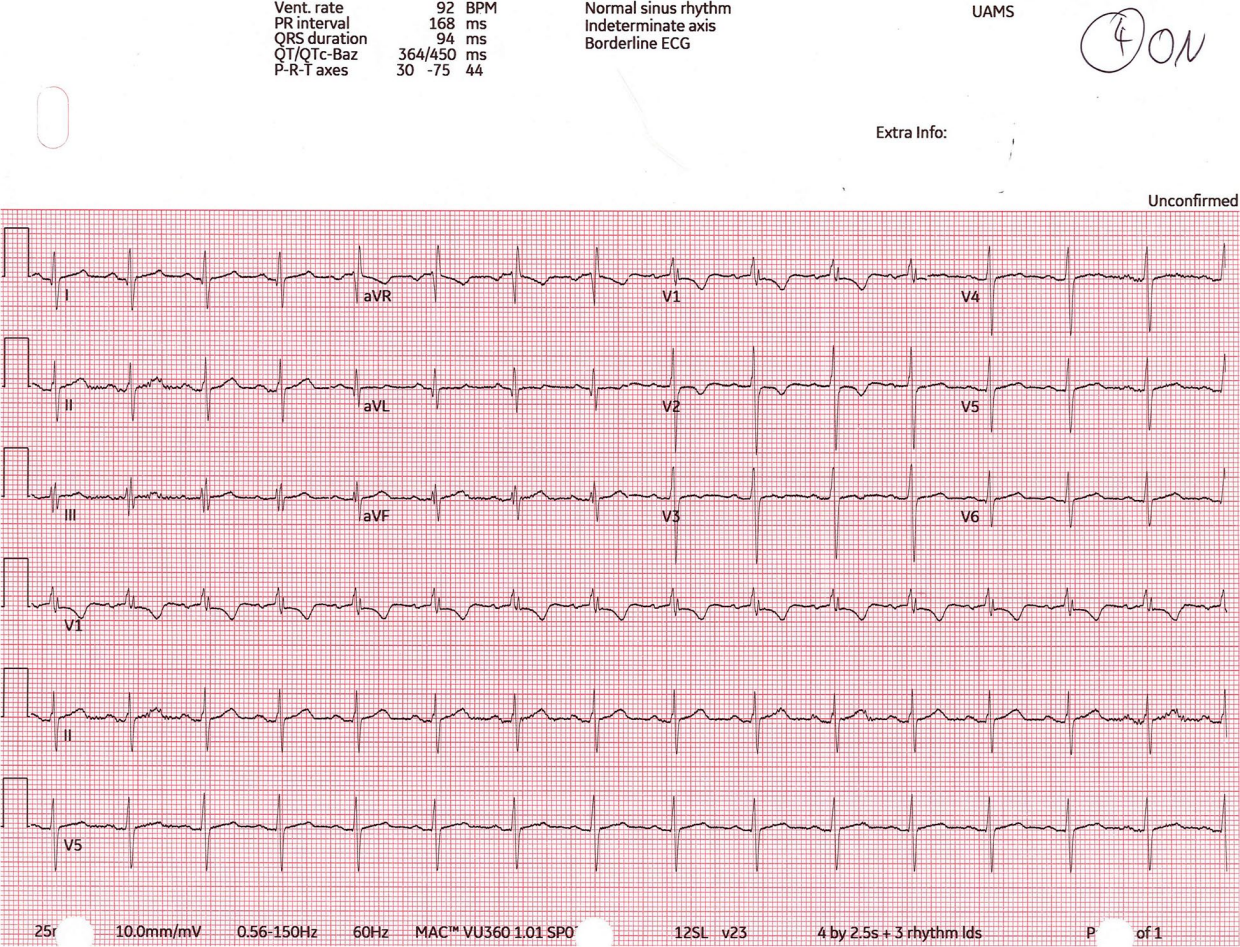
Postoperative ECG, 12 months after surgery, the normal VNS output was gradually increased from 0 mA to 0.75 mA without any significant bradyarrhythmic changes

We present a 28-year-old male patient with a history of Asperger's syndrome, moderate mental retardation, disruptive behavior disorder, and pharmacoresistant epilepsy. The patient’s first seizure occurred at three months old without any obvious cause. Brain MRI studies were without abnormalities. Ambulatory EEG recordings demonstrated generalized, 2–4 Hz epileptiform discharges seen throughout the various stages of wakefulness, drowsiness, and sleep; in addition, there were numerous generalized electrographic seizures of the same 3–4 Hz frequency suggesting absence type seizures. The patient was experiencing five to six generalized clonic-tonic seizures a week despite numerous medication trials; thus, VNS was implanted seven years ago. The VNS therapy was highly effective and the patient experienced only two seizures since the surgery until a year ago. Since then, the patient has had three episodes of generalized clonic-tonic seizures. Additionally, two months ago the patient began experiencing a stabbing pain at the IPG site four to five times a week, lasting up to 5 min. All this was suspicious for VNS system malfunction, but device interrogation was not concerning – it showed the Demipulse device (model 103) with normal output current set at 1.5 mA with 30 Hz frequency and 500 ms pulse width at 16% duty cycle (30 s on, 3 min off) with remaining battery 8–18% and with 2746 Ohm system impedance. A CT of the neck and chest was obtained and showed unusually tilted IPG but no obvious hardware or surrounding tissue problems (Fig. 2). Additionally, plain radiographs were obtained and showed VNS contacts at C5 level without visible hardware discontinuities (Fig. 3). Breakthrough seizures and stabbing pains prompted the consideration of VNS system exploration surgery. Worth noting, the patient had incomplete right bundle branch block on preoperative ECG (Fig. 4), thus, preoperative cardiology evaluation was requested. However, our patient did not follow with this recommendation. Due to the fact that our patient already had VNS in place and it was highly effective, the decision was made to proceed with surgery. During the surgery the new IPG was implanted—AspireSR (model 106). The close inspection of the original VNS lead in the chest pocket revealed the ruptured insulation layer. Thus, the surgery was extended to the VNS lead replacement to the new PerenniaFLEX lead (model 304–30). Noteworthy, the new lead contacts were placed more distally (caudally) on the freshly dissected segment of the left vagus nerve as the old contacts were embedded in the tight epineurial scar and, thus, were left in situ. However, the intraoperative device interrogation with a standard 1 mA output resulted in immediate asystole for the duration of stimulation and it was reproducible until the output was decreased to 0.675 mA which is less than a half of the original output value (1.5 mA). In the postanesthesia recovery room, the patient was experiencing excessive coughing abolishable by prolonged magnet application over IPG. Therefore, further programming was performed with normal output current titration down to 0.25 mA, with signal frequency of 20 Hz, pulse width of 250 ms, on time of 30 s, and off time of 2 min with AutoStim output turned off. At 6 months after surgery, the normal output current was titrated to 0.5 mA without side effects. At 12-month postoperative follow up, the patient’s caregiver reported “small staring spells once every few days but no big seizures since surgery”. The plain radiograph of the neck showed the retained old VNS contacts at C5 level and the new VNS contacts more caudally (Fig. 5). The upward titration of VNS output was arranged with live concomitant ECG, IV access, and atropine available. First, VNS was turned off and ECG was recorded to establish the baseline (Fig. 6). Then the normal VNS output was gradually increased to 0.75 mA without any significant bradyarrhythmic changes on ECG (Fig. 7). Further titration to 1 mA resulted in excessive coughing, thus, it was reverted to 0.75 mA. Three months later, the patient was seen again and VNS normal output was successfully increased to 1.0 mA without any significant ECG changes or clinical side effects.

## Discussion and conclusions

To the best of our knowledge, this is the first case report of immediate asystole associated with surgical VNS lead replacement. Our case highlights the possibility of new severe cardiac bradyarrhythmias following surgical VNS lead replacements even in patients without preoperatively known clinical cardiac side effects. Although VNS-related bradyarrhythmic complications are rare, they can result in detrimental morbidity if not promptly recognized and addressed. On a technical note, when performing VNS system diagnostics, which includes stimulation circuit impedance measurement, with device models from M103 to 1000, the system will run the test at the output current that it is currently programmed at; the exception is when the current output is set to 0 mA, such as new VNS IPGs in the operating room – then the system testing will be run at 1 mA. The older device models (M102R and earlier) will always run system diagnostics at 1 mA for 5 s. In our case, the observed reproducible asystole corresponded with vagal nerve stimulation at or above 0.75 mA whereas preoperatively the patient did not exhibit symptomatic bradyarrhythmia with VNS output set at 1.5 mA. Similar accounts of cardiac complications such as bradyarrhythmia and asystole have been reported in patients who underwent VNS implantation or VNS IPG replacement surgeries, further illuminating the significance of being aware of these potential complications [5, 9–11]. However, unlike the previous reports that have reported late onset arrhythmias, our case showed immediate intraoperative asystole corrected by decreasing the VNS output current below 0.75 mA.

We speculate that our patient developed the cardiac right bundle branch block at some time after the VNS system malfunction; the stimulation current leak (pain at IPG site) precluded effective vagus nerve stimulation with resultant breakthrough seizures, but also prevented the manifestation of asystole. The successful vagal nerve re-challenge at 0.75 mA one year after surgery could be explained by vagal-cardiac habituation to chronic stimulation effects.

Regarding surgical techniques concerning VNS revision surgeries, there are nuances and variations among experts. For example, Giordano et al. [12] mention the use of electrical cautery and attempts to remove old VSN lead components in every case. We are particularly concerned about thermal and mechanical nerve damage, therefore, we do not use monopolar cautery during any of VNS surgeries as it seems not necessary. We also do not use bipolar cautery within carotid sheath, i.e. in proximity to vagus nerve. We also do not attempt to remove helical contacts from the nerve as long as remaining lead segments are less than 2 cm long as it does not interfere with subsequent MRI acquisitions in MRI exclusion zones [13, 14].

Based on our and published experience, we suggest the following set of measures to minimize the risk of perioperative cardiac arrhythmic complications related to vagus nerve stimulation:

1. the preoperative electrocardiography followed by cardiology evaluation for detected abnormalities for all patients undergoing new VNS implantations, as well as revision surgeries for VNS malfunctions;
2. the surgeon should aim to place the electrodes as distally on the vagus nerve as technically possible in order to avoid stimulation of the cardiac branches; at the same time staying within carotid sheath to avoid vagus-look-alike nerves such as the phrenic nerve and ansa cervicalis;
3. the VNS should be implanted on the left vagus nerve whenever possible to avoid cardiac side effects; however, if VNS implantation on the right vagus nerve results in an acceptable outcome, that approach may be considered in specific cases [12];
4. if patient is being operated for VNS malfunction, use lower current output for system diagnostics and stimulation resumption than the original patient settings;
5. whereas the additional non-routine intraoperative monitoring techniques seem unnecessary, the anesthesia team should be alerted about the start of VNS system diagnostics and remain vigilant of cardiac rhythms to be prepared for the immediate management
6. if patient develops asystole during system diagnostics, there is no other option but to pull the VNS lead out from IPG receptacle. Therefore, for the system diagnostics, we recommend always keeping the IPG outside the patient and the setscrew driver inside the setscrew head in case the lead removal needs to be done quickly or, even better, not securing setscrew at all. Of note, the VNS wand can be kept on the other (nonlabeled) side of IPG during interrogation in order not to interfere with the screwdriver if the surgeon prefers to keep the setscrew tightened. Then, for the second phase of testing for devices with AutoStim capability (models 106 and later), the IPG needs to be placed inside the subcutaneous pocket to verify the correct heart rate detection;
7. intraoperative bradycardia alone during VNS surgery should not be a reason to abort the procedure, however, the onset and titration of VNS therapy should be monitored with simultaneous ECG [15].

## Data Availability

Not applicable

## Abbreviations

VNS: Vagal nerve stimulation
FDA: Food and Drug Administration
ECG: Electrocardiogram
IPG: Implantable pulse generator
